# The Bite of the Rattlesnake

**Published:** 1848-02

**Authors:** Chas. A. Phelps


12. *The Bite of the Rattlesnake.* By CHAS. A. PHELPS, M. D. (Communicated for the Boston Medical and Surgical Journal.)

The fate of the late lamented Dr. Wainwright, of New York, gives at this time a sad interest to inquiries on this subject. In the account of his case published in the newspapers, the full details of his treatment are not given. There is one remedy, however, which appears deserving of further trial. I refer to olive oil.

In the Philosophical Transactions of the Royal Society of London, for the year 1734, mention is made of a viper catcher, who having been frequently bitten, had always cured himself with sweet olive oil. He was induced to make trial of its effects at a meeting of the Royal Society. Stripping his arm, he compelled the enraged animal to strike him forcibly. The poison was allowed to act upon his system until his head, face and tongue were greatly swollen, his face and arm quite black, and his senses much affected. Oil was then given internally, and the wound freely bathed with the same, after which he gradually but soon recovered. In the same volume an account is furnished of some experiments made subsequently at Oxford, in which a viper could not be made to bite a part of the hand which had been smeared with oil, although it did so readily after the oil was removed. These undoubtedly were the common English vipers—the coluber berus of Linnæus.

In Vol. II., No. 2, of the Medical Repository, published in New York in 1798, an article is found narrating its use in South Carolina in 1786, in the case of a woman bitten by the deadly rattle-snake of our country (the crotalus of Linnæus.) In this instance the head and face were greatly swollen, the tongue swollen and protruded, the face black, the senses affected, and extreme difficulty in respiration. Two drachms of olive oil were administered internally, followed by an immediate abatement of the symptoms, and in thirty minutes by emesis and dejections. After this she became rapidly convalescent, and soon wholly recovered.

To come nearer home, I would mention a case related to me several years since by Dr. A. Phelps, of this city. It was that of a man who had some fifty rattlesnakes which he exhibited. Imprudently exposing himself on one occasion, he was severely bitten in the hand. The usual symptoms immediately manifested themselves. Olive oil was given internally, and the hand and wrist immersed in the same for twelve hours. In a short time after the oil was exhibited, the symptoms subsided, and the following day the man was as well as usual.

This remedy was used successfully at Dresden by Dr. Vater. Also in England by Mr. Oliver (for the history of his experiments, see Philosophical Transactions, Volume XXXIX). It is said to have been used ineffectually at Paris by Messrs. Geoffroy and Hunauld, of the Royal Academy. Combined with ammonia it was highly recommended by the celebrated Bernard de Jussieu. Dr. Mead tells us that the viper catchers in England used, as a specific upon which they placed the greatest reliance, the *axungia!* of the viper rubbed into the wound. The ointment of M. Gondret was prepared with oil of olives, ℥ ss.; tallow, ℥ ss.; ammonia, ℥ j. Orfila, in his work on Poisons, recommends the application of heated olive oil to the wound. The famous *eau de luce*, which was attended with success in the hands of de Jussieu and M. Sonnini, the latter of whom, in his Travels in Greece and Turkey, details an interesting case of a child cured by its use, is well known to have been composed of oleum succini in union with a volatile alkali. Is it not probable that these remedies acted in a similar manner to olive oil itself?

It is not necessary to speak here of the various other remedies advised in the treatment of these venomous bites. It is to be regretted that opinions on this subject are so unsettled, and that more satisfactory results have not been always reached. I would ask, however, if the foregoing does not warrant a further use of olive oil. Whether any resort was had to it in the case of Dr. W. before alluded to, I am not informed. Boston, January 14, 1848.—*Boston Med. and Surgical Journal.*

